# Allosteric Cannabinoid Receptor 1 (CB1) Ligands Reduce Ocular Pain and Inflammation

**DOI:** 10.3390/molecules25020417

**Published:** 2020-01-20

**Authors:** Dinesh Thapa, Elizabeth A. Cairns, Anna-Maria Szczesniak, Pushkar M. Kulkarni, Alex J. Straiker, Ganesh A. Thakur, Melanie E. M. Kelly

**Affiliations:** 1 Department of Pharmacology, Dalhousie University, Halifax, NS B3H 4R2, Canada; 2 Department of Pharmaceutical Sciences, Northeastern University, Boston, MA 02115, USA; 3 Department of Psychological and Brain Sciences, Indiana University, Bloomington, IN 47405, USA; 4 Department of Anesthesia, Pain Management & Perioperative Medicine, Dalhousie University, Halifax, NS B3H 4R2, Canada

**Keywords:** cannabinoids, allosteric ligands, cornea, pain, inflammation

## Abstract

Cannabinoid receptor 1 (CB1) activation has been reported to reduce transient receptor potential cation channel subfamily V member 1 (TRPV1)-induced inflammatory responses and is anti-nociceptive and anti-inflammatory in corneal injury. We examined whether allosteric ligands, can modulate CB1 signaling to reduce pain and inflammation in corneal hyperalgesia. Corneal hyperalgesia was generated by chemical cauterization of cornea in wildtype and CB2 knockout (CB2^−/−^) mice. The novel racemic CB1 allosteric ligand GAT211 and its enantiomers GAT228 and GAT229 were examined alone or in combination with the orthosteric CB1 agonist Δ^8^-tetrahydrocannabinol (Δ^8^-THC). Pain responses were assessed following capsaicin (1 µM) stimulation of injured corneas at 6 h post-cauterization. Corneal neutrophil infiltration was also analyzed. GAT228, but not GAT229 or GAT211, reduced pain scores in response to capsaicin stimulation. Combination treatments of 0.5% GAT229 or 1% GAT211 with subthreshold Δ^8^-THC (0.4%) significantly reduced pain scores following capsaicin stimulation. The anti-nociceptive effects of both GAT229 and GAT228 were blocked with CB1 antagonist AM251, but remained unaffected in CB2^−/−^ mice. Two percent GAT228, or the combination of 0.2% Δ^8^-THC with 0.5% GAT229 also significantly reduced corneal inflammation. CB1 allosteric ligands could offer a novel approach for treating corneal pain and inflammation.

## 1. Introduction

The cornea has one of the densest concentrations of unmyelinated sensory nerve endings in the body [1,2], which are highly sensitive to mechanical stimulation, temperature, and various chemicals mediators, through receptors such as the transient receptor potential family [3]. Damage or irritation to these nerve endings resulting from ocular surface manipulations such as cataract surgery [4], long-term and improper use of contact lenses [5,6,7], and frequent exposure to irritating environmental and chemical stimuli (infection, air pollutants, hazardous chemicals, air pressure etc.) [1,8,9], can lead to local release of inflammatory mediators, including calcitonin gene-related peptide (CGRP), causing corneal inflammation [10]. Stimulation of corneal nerves following damage can result in ocular pain, either to normally non-noxious stimuli (allodynia) and/or as a heightened pain response to noxious stimuli (hyperalgesia) [9,11,12,13], which can result in sensitization and neuropathic pain over time [14]. Current pharmacotherapies for corneal pain and inflammation include topical steroids, non-steroidal anti-inflammatory drugs, antibiotics, as well as other agents for neuropathic pain including, tricyclic antidepressants, GABAergic drugs (e.g., gabapentin), opioids, etc. [12,15,16]. However, these treatments are not always effective enough to produce adequate pain relief, especially where both pain and inflammation may need to be controlled [13,15,17].

Modulation of the endocannabinoid system (ECS) has emerged as a novel approach to treat pain and inflammation, among other conditions [18,19,20]. The ECS is comprised of G protein-coupled receptors (cannabinoid receptor 1, CB1; and cannabinoid receptor 2, CB2), endocannabinoids, and the enzymes responsible for their synthesis and degradation [21,22,23,24,25,26,27]. Support for a functional role for the ECS, and specifically CB1 activation in modulating TRPV1-mediated corneal pain, was provided by a recent paper demonstrating that cannabinoids that act at CB1, such as tetrahydrocannabinol (THC), are able to reduce corneal hyperalgesia to a capsaicin challenge and, additionally, also produce a reduction in corneal injury-induced inflammation [28]. 

**Figure 1:**
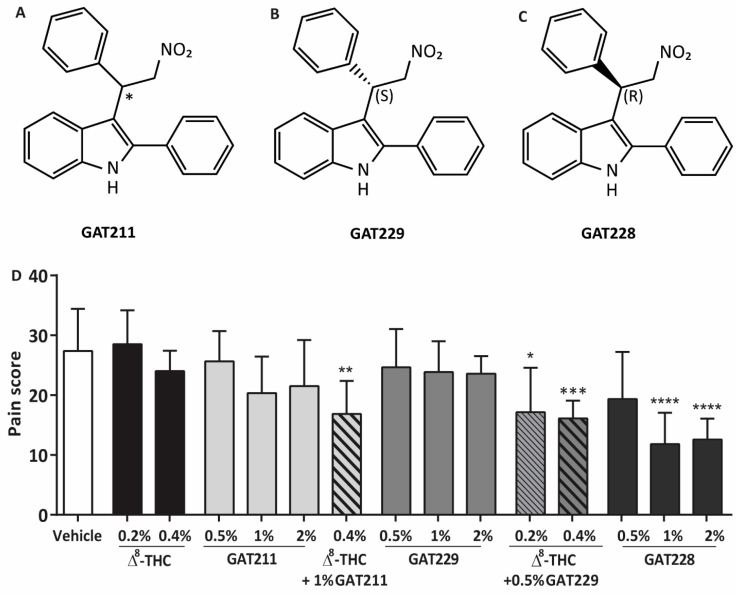
The structure of (**A**) GAT211, the (**B**) *R*-(+)-enantiomer GAT228 and the (**C**) *S*-(−)-enantiomer GAT229 [34]. (**D**) Dose-response for GAT211 (0.5–2.0%, *n* = 6 per group), GAT229 (0.5–2%, *n* = 6 per group) and GAT228 (0.5–2%, *n* = 5–7 per group) following capsaicin challenge. Topical administration of GAT211 or GAT229 in combination with 0.4% Δ^8^-THC, or GAT228 alone or reduces corneal hyperalgesia in WT mice following cauterization. Values represent mean ± SD. For statistical analysis, one-way ANOVA with Dunnett’s post hoc test (compared to vehicle) was used. * *p* < 0.05, ** *p* < 0.01, *** *p* < 0.001, **** *p* < 0.0001.

However, clinical usefulness of CB1 agonists, particularly in chronic applications, may be limited due to desensitization, tolerance, and behavioral side-effects [29,30,31]. Drugs that target an allosteric binding site at CB1 could offer a novel approach to modulate CB1 and reduce corneal pain, enabling the long-term use of drugs that may provide more favorable receptor kinetics and side-effect profiles [32,33]. GAT211 (Figure 1A) and its enantiomers GAT228 (*R*; Figure 1C), and GAT229 (*S*; Figure 1B) are a group of novel compounds that have been reported to modulate CB1 through the allosteric site [34]. GAT211 was reported to have both allosteric agonist and positive allosteric modulator (PAM) activity at CB1; the *R*-(+)-enantiomer GAT228 was demonstrated to have partial allosteric agonist activity, while the *S*-(−)-enantiomer GAT229 was shown to behave as a clean PAM [34]. Additionally, in neuropathic and inflammatory models of pain, GAT211 when delivered alone reduced allodynia without producing typical CB1-mediated cannabimimetic side-effects [35], as did a related CB1 PAM, ZCZ011 [36]. In addition, unlike the orthosteric CB1 agonist WIN55,212-2, GAT211 did not produce tolerance over a 19 day interval of once-daily dosing [35]. 

This paper explored the potential for CB1 modulation by the CB1 allosteric ligands GAT211, GAT228 or GAT229, alone or in combination with the CB1 orthosteric agonist Δ^8^-tetrahydrocannabinol (Δ^8^-THC), in a mouse model of chemical injury-induced corneal hyperalgesia.

## 2. Results

### 2.1. GAT211 and GAT229 Potentiated the Anti-Nociceptive Effects of Δ^8^-THC, Whereas GAT228 Directly Reduced Corneal Pain 

Different concentrations of the racemic compound GAT211, and the resolved enantiomers GAT229 and GAT228, were applied topically in WT mice to establish the effective concentrations required to reduce the corneal pain score compared to the vehicle-treated group (27 ± 7, *n* = 8; Figure 1D). Some compounds were then tested in combination with subthreshold concentrations of Δ^8^-THC. Administration of 0.4% Δ^8^-THC did not reduce the pain score in capsaicin-challenged corneas (*p* > 0.05, *n* = 6) as previously reported [28], nor did administration of 0.2% Δ^8^-THC (*p* > 0.05, *n* = 6).

For the racemic compound GAT211, we tested topical concentrations of 0.5%, 1%, or 2% but none of these concentrations were effective in reducing corneal pain compared to vehicle-treated eyes (*p* > 0.05, *n* = 6 per group). Topical treatment of animals with 0.4% Δ^8^-THC with 1% GAT211 significantly reduced the corneal pain score compared to vehicle-treated eyes (17 ± 6, *p* < 0.01, *n* = 6). Likewise, topical application of GAT229 (0.5%, 1%, or 2%, *n* = 6–7 per group) alone did not reduce corneal pain (*p* > 0.05), but the combination of 0.2% Δ^8^-THC or 0.4% Δ^8^-THC with 0.5% GAT229 significantly reduced the corneal pain response (17 ± 7 and 16 ± 3, respectively) compared to vehicle-treated eyes (*p* < 0.05 and *p* < 0.001, *n* = 6 and 10, respectively). For GAT228, mice receiving 0.5% GAT228 (*n* = 6) did not have a significant reduction in pain score compared to vehicle-treated mice (*p* > 0.05). Increasing the concentration of GAT228 to 1% and 2%, unlike GAT211 or GAT229, did significantly reduce the pain score (12 ± 5 and 12 ± 4, *p* < 0.0001, *n* = 6 and 7, respectively).

### 2.2. GAT229 and GAT228 Reduce Corneal Pain via Activation of CB1

**Figure 2:**
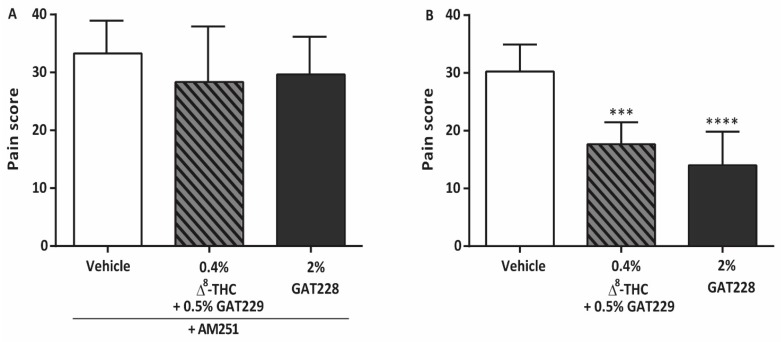
The antinociceptive effects of GAT229 and GAT228 are blocked by antagonism of CB1 by AM251 (2.0 mg/kg i.p.). (**A**) Pain score measured at 6 h post-cauterization and following administration of 5 μL of topical vehicle, 0.4% Δ^8^-THC + 0.5% GAT229, or 2% GAT228 (*n* = 6–7 per group) in WT mice pre-administered with AM251 (**B**) Pain score measured in CB2^−/−^ mice following administration of 5 μL of topical vehicle, 0.4% Δ^8^-THC + 0.5% GAT229 or 2% GAT228 (*n* = 6–8 per group). Values represent mean ± SD. For statistical analysis one-way ANOVA with Dunnett’s post hoc test (compared to vehicle) was used. *** *p* < 0.001, **** *p* < 0.0001.

To verify that these effects occurred through a CB1-dependent mechanism, the CB1 antagonist AM251 (2.0 mg/kg, i.p.), was administered prior to corneal cauterization and capsaicin stimulation. In mice receiving AM251, the anti-nociceptive actions of 0.4% Δ^8^-THC plus 0.5% GAT229 (28 ± 10, *n* = 6) were not significantly different compared to vehicle-treated eyes plus AM251 (33 ± 6, *p* > 0.05, *n* = 7, Figure 2A), indicating that the actions of Δ^8^-THC plus 0.5% GAT229 are mediated via CB1. Likewise, the anti-nociceptive effects of 2% GAT228 are absent in mice pre-treated with CB1 antagonist AM251 (30 ± 7, *n* = 6) compared to vehicle-treated eyes plus AM251 (*p* > 0.05, Figure 2A). Figure 2B shows the pain score measured in cauterized eyes in CB2^−/−^ mice following treatment with vehicle, 0.4% Δ^8^-THC plus 0.5% GAT229, or 2% GAT228. Both 0.4% Δ^8^-THC plus 0.5% GAT229 and 2% GAT228 reduced the corneal pain score (18 ± 4 and 14 ± 6, respectively, *n* = 6 in each group) compared to vehicle-treated eyes (30 ± 5, *p* < 0.001 and *p* < 0.0001, respectively, *n* = 8), suggesting that the GAT-mediated reduction of corneal pain seen with GAT229 with Δ^8^-THC and GAT228 is independent of CB2. 

### 2.3. GAT229 in Combination with Δ^8^-THC, and GAT228 Alone, Reduced Neutrophil Infiltration to the Cornea

**Figure 3:**
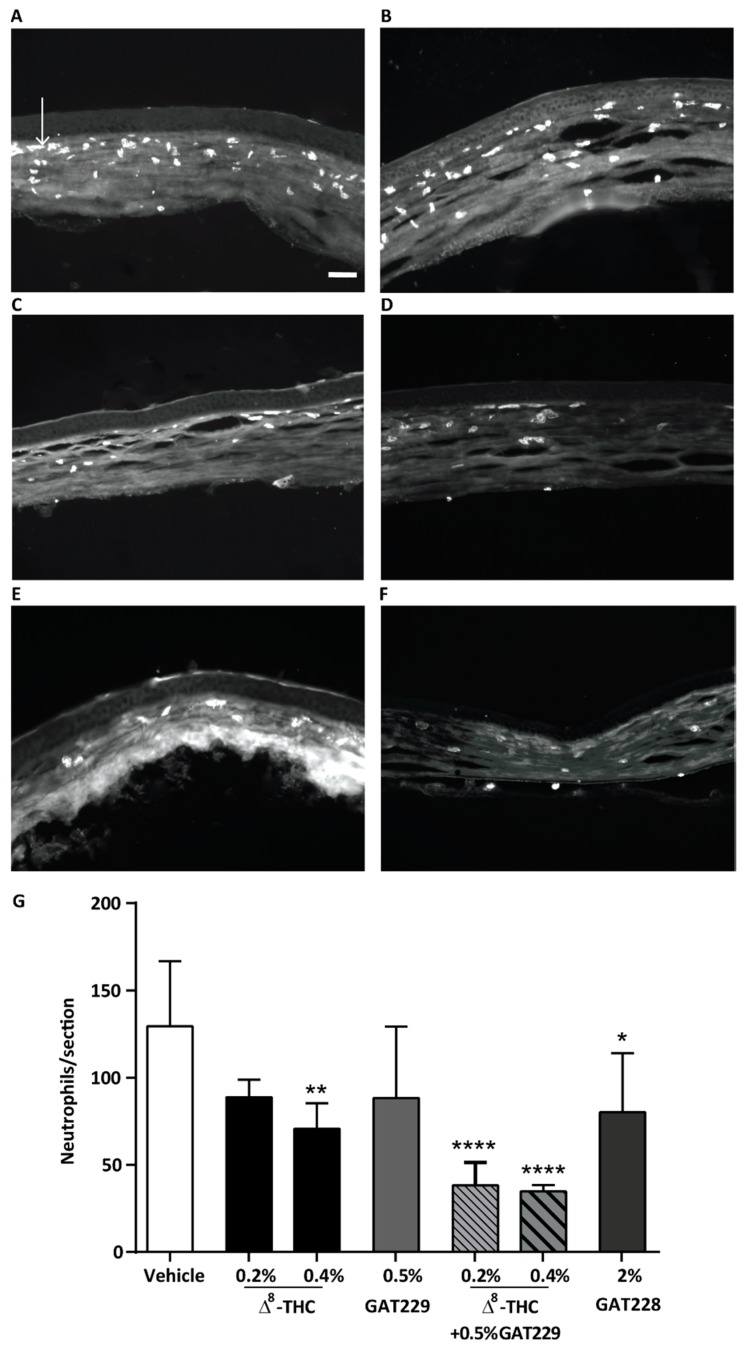
Neutrophil expression in cauterized corneas at 6 h post injury following the topical treatments of drug or vehicle and capsaicin stimulation. Representative images of transverse sections of the central cornea from (**A**) vehicle-treated corneas, (**B**) 0.2% Δ^8^-THC-treated corneas, (**C**) 0.5% GAT229-treated corneas, (**D**) 2% GAT228-treated corneas and (**E**) 0.2% Δ^8^-THC + 0.5% GAT229, (**F**) 0.4% Δ^8^-THC + 0.5% GAT229 and (**G**) effects of topical treatment of WT cauterized eyes with (0.2 and 0.4%) Δ^8^-THC, 0.5% GAT229, 2% GAT228 or, 0.2% or 0.4% Δ^8^-THC + 0.5% GAT229 (*n* = 4–6 per group) in neutrophil infiltration compared to vehicle-treated eyes (*n* = 7). Values represent mean ± SD. Arrow in (**A**) points to one of many infiltrating neutrophils. Scale bar: 50µm. For statistical analysis one-way ANOVA with Dunnett’s post hoc test (compared to vehicle) was used. * *p* < 0.05, ** *p* < 0.01, **** *p* < 0.0001.

Neutrophil infiltration into the cornea of WT mice was examined at 6 h following treatment with topical vehicle, Δ^8^-THC (0.2% and 0.4%), 0.5% GAT229, 2% GAT228, or 0.2 or 0.4% Δ^8^-THC plus 0.5% GAT229. In WT mice, topical treatment of 0.2% Δ^8^-THC did not reduce neutrophil infiltration compared to vehicle-treated eyes (*p* > 0.05, *n* = 6 per group, Figure 3). However, 0.4% Δ^8^-THC significantly reduced neutrophil infiltration (71 ± 15, *n* = 6) compared to vehicle-treated group (130 ± 37, *p* < 0.01, *n* = 7). Administration of 0.5% GAT229 alone also did not result in reduced neutrophil infiltration compared with vehicle (*p* > 0.05, *n* = 5), though, combination of 0.2% Δ^8^-THC with 0.5% GAT229 significantly reduced neutrophil infiltration (38 ± 13, *n* = 4) compared to vehicle-treated group (*p* < 0.0001), as did combination of 0.4% Δ^8^-THC with 0.5% GAT229 (35 ± 4, *p* < 0.0001). Administration of 2% GAT228 also significantly reduced neutrophil infiltration (80 ± 34, *n* = 5) compared to vehicle-treated eyes (*p* < 0.05, Figure 3G).

## 3. Discussion

Hyperalgesia is a well-documented symptom of inflammatory and/or neuropathic pain [13,37,38,39]. In our mouse model of corneal injury, we observed a heightened pain response (hyperalgesia) to chemical stimuli. Previous studies have shown that activation of CB1 reduces TRPV1-mediated pain and inflammation in other models of pain, including nerve growth factor-sensitized pain [40,41], as well as inflammation-induced pain in urinary bladder [42]. In the cornea, we have previously reported that activation of CB1 by Δ^8^-THC reduces a TRPV1-induced corneal pain response, provoked through a capsaicin challenge, and reduces neutrophil infiltration [28]. Consistent with our findings, CB1 activation in cornea has been implicated in TRPV1 desensitization and a decrease in pro-inflammatory mediators after corneal injury [20]. CB1 has also been reported to be important for the normal course of corneal wound healing [20,43]. Therefore, together with previous data [28], we have further provided evidence to support that activation CB1 may be a good target for corneal neuropathic pain management by direct modulation of the sensation of pain, and the inflammatory response which may lead to sensitization over time.

However, therapeutic use of CB1 orthosteric agonists may be limited due to side-effects such as dose-dependent receptor desensitization, and off-target effects [30,31,32,44,45]. CB1 positive allosteric modulators may be advantageous in that they may provide an alternate means to modulate CB1, but with fewer of these limitations. PAMs may stabilize receptor conformations in such a way that they can fine-tune the effects of orthosteric ligands [46,47], increasing affinity and/or efficacy of binding, and ultimately resulting in changes in downstream signaling [33,44,48,49]. Previous studies have reported that administration of GAT211 and ZCZ011, a related CB1 PAM, reduced mechanical and cold hyperalgesia in mouse models of neuropathic and inflammatory pain [35,36]. GAT211 treatment did not show evidence of anti-nociceptive tolerance throughout the entire 19 day dosing period, or physical dependence (measured by paw tremors) at 20 days following the treatment [35]. Similarly, there was no difference in the anti-nociceptive effect of ZCZ011 following 6 days of chronic dosing (40 mg/kg, i.p. b.i.d) compared to acute dosing at day 1 (40 mg/kg, i.p.) [36]. Administration of GAT211 alone also did not produce side-effects associated with orthosteric activation of CB1, such as those produced by Δ^9^-THC, or WIN55,212-2 [35].

In this paper, we have shown that topical application of the racemic ago-PAM GAT211, or the PAM GAT229, potentiated the corneal anti-nociceptive effects of a subthreshold dose of the orthosteric agonist Δ^8^-THC. Additionally, GAT229 also potentiated the anti-inflammatory effects of a subthreshold dose Δ^8^-THC measured at 6 h following capsaicin stimulation. Dose-dependent potentiation of cannabimimetic side-effects with the combination of CB1 PAMs with orthosteric agonists, however, was reported by both Slivicki [35] and Ignatoawaska-Jankowska [35] and their colleagues. Therefore, avoiding administration of the combination of CB1 PAMs with exogenous orthosteric agonists may be advisable. Local increases of endocannabinoids at the site of pathology that are enough to potentate the actions of CB1 PAMs may be more desirable as a treatment paradigm; it would enable PAMs to be administered without requiring the addition of orthosteric agonists. Such was the case in models of neuropathic and inflammatory pain treated with GAT211 and ZCZ011 [35,36], and for intraocular pressure lowing in a mouse model of ocular hypertension, an effect which was absent in normotensive mice [50]. In our corneal hyperalgesia model, administration of GAT211 or GAT229 reduced corneal pain when combined with subthreshold Δ^8^-THC, but not on their own. This may be due to lack of, or insufficient, local endocannabinoid production to allow PAMs to potentiate activity at CB1, at least at the time point used to measure the pain response in this study. Unlike previous studies investigating the in vivo effects of CB1 PAMs, our model is relatively acute, with drug administration occurring shortly after cauterization, and capsaicin challenge occurring only 6 h later. In contrast, AEA and 2-AG were significantly increased in the brain and spinal cord 3 and 7 days following injury [51], and at least 14 days in the dorsal root ganglia [52], consistent with the timeline of effects observed with PAM administration [36]. Further studies using a more chronic model of corneal pain would therefore be useful to investigate if increases in endocannabinoid levels following injury in the cornea are sufficient to permit PAM potentiation of CB1 activation by endocannabinoids. Although avoiding PAM-potentiated psychoactivity may not be an issue with respect to topical administration to the eye, it has been reported that following ocular topical administration, WIN 55,212-2 can be detected in the urine [53]. While psychoactivity from this route of administration has not been reported, chronic application over time of exogenous potent CB1 agonists may still have the potential to result in systemic drug levels. Therefore, use of lower doses of CB1 agonists together with a PAM for topical use may also have advantages to avoid systemic side-effects.

Finally, unlike GAT211 or GAT229, the allosteric agonist GAT228, significantly reduced corneal pain and inflammation on its own, consistent with actions of an allosteric agonist, as previously reported *in vitro* [34]. While the actions of allosteric agonists at CB1 have yet to be fully characterized, it is possible that activation through the allosteric site may retain some advantages of allosteric modulators over orthosteric agonists [54,55]. However, it was recently suggested that in absence of a ligand at the orthosteric site, GAT228 may bind with equal affinity to either the allosteric or orthosteric site [56]. While we cannot exclude that the actions of GAT228 are due to orthosteric rather than allosteric agonism, in radioligand binding experiments GAT228 (up to 1 µM) did not displace the orthosteric agonist, implying allosteric interactions [34]. Further investigation of chronic dosing of GAT228 could be worthwhile and may identify if use of this compound could provide benefit over more traditional orthosteric agonists.

This paper provides further evidence supporting the role of CB1 in modulating capsaicin-evoked corneal pain responses and inflammation. Here, we present for the first-time evidence that allosteric activation of CB1 using the PAMs GAT211 or GAT229, in combination with subthreshold dose of CB1 orthosteric agonist Δ^8^-THC, or the CB1 ligand GAT228 alone, reduced both corneal pain and inflammation. CB1 PAMs in combination with subtherapeutic dose of orthosteric agonists, or CB_1_ allosteric agonists alone, could be a novel approach for the treatment of corneal pain and inflammation.

## 4. Materials and Methods

### 4.1. Experimental Animals

All animal experiments and care complied with the Canadian Council for Animal Care guidelines (http://www.ccac.ca/) and the ARVO Statement for the Use of Animals in Ophthalmic and Vision Research. Protocols were approved by the Dalhousie University Committee on Laboratory Animals or by the Indiana University Animal Care Committee.

Male BALB/c WT (25–30 g; Charles River Laboratories, Saint Constant, QC, Canada), age matched (8–12 weeks) CB2 knockout mice (CB2^−/−^), and adult C57Bl/6 (Envigo, Indianapolis, IN, USA) were used in this study. CB2^−/−^ mice used in this study were produced by crossing male C57BL/6J CB2^−/−^ mice (strain B6.129P2-Cnr2tm1Dgen/J; Jackson Laboratory, Bar Harbor, ME, USA) with inbred BALB/c female mice (Charles River Laboratories, Saint Constant, QC, Canada) for ten generations. CB2^−/−^ was confirmed via genotyping as described by Thapa et al. [28]. Animals were kept on a 12 h light/dark cycle with unrestricted access to food and water.

### 4.2. Induction of Corneal Injury

Corneal injury in mice was induced as previously described in Thapa et al. (2018), using a protocol adapted from a rat model of corneal hyperalgesia [57]. Briefly, mice were anesthetized using 2–3% isoflurane and corneal injury was induced in both eyes using a silver nitrate-coated (MedPro^®^, 75% silver nitrate, 25% potassium nitrate; AMG Medical Inc., Montreal, QC, Canada) micro-applicator brush (Centrix Inc., Shelton, CT, USA). The micro-brush was held in contact with the cornea for 2 s, producing a distinct superficial white lesion (~1 mm diameter) in the epithelial cell layer. Cauterized eyes were then rinsed several times with room temperature saline. An ocular lubricant (Systane^®^, Alcon Canada Inc., Dorval, QC, Canada) was applied to the corneal epithelial surface to reduce corneal drying. Mice recovered fully from anesthesia within 3–5 min post-cauterization.

### 4.3. Assessment of Behavioral Pain Response

Corneal sensitivity to an acute capsaicin stimulus was evaluated in animals at six hours following cauterization injury, as previously described [28]. Mice were lightly restrained and injured eyes were challenged with capsaicin (1 µM) stimulation applied topically (5 µL). Each animal was given a single dose of capsaicin per eye (right eye first, followed by left eye) to elicit a pain response and to avoid the desensitization which may occur from repeated application. Topical application of capsaicin was associated with rapid blinking that occurred over a 30 s period and was accompanied by occasional eye wiping. The number of blinks and eye wipes were used as an indicator of ocular pain, as previously described [28]. Pain behaviors were recorded in the tested eyes using an iPhone 5S (8 megapixel). Offline analysis was carried out by an experimenter blinded to the treatments given. Videos were analyzed offline in slow motion (play speed 0.5, Windows Media Player version 10) and the pain response was scored by adding the total number of blinks and eye wipes recorded in each eye over the entire 30 s recording period following capsaicin application to give a composite pain score.

### 4.4. Neutrophil Migration

Following behavioral pain assessments at 6 h, mice were sacrificed, and eyes were enucleated and fixed in 4% paraformaldehyde followed by 30% sucrose overnight. Corneal sections (12 µm) were prepared using a CM1850 cryostat (Leica, Wetzlar, Germany). Sections were washed for 4 times in phosphate-buffered saline (PBS; Sigma-Aldrich, Oakville, ON, Canada), blocked for non-specific binding (10% normal goat serum in 0.5% Triton-X/PBS, Sigma-Aldrich, Oakville, ON, Canada) for 2 h, followed by incubation for 2 nights at 4 °C in purified rat-anti Ly-6G antibody (1:200 in 0.5% Triton-X/PBS; Abcam, Cambridge, MA, USA). Sections were then washed with PBS 4 times for 10 min, followed by an overnight incubation with the secondary antibody (1:500, goat anti-rat Alexa Fluro^®^ 488, Jackson ImmunoResearch Laboratories, Inc., West Grove, PA, USA). Stained sections were then washed 4 times with PBS and mounted on Superfrost slides (Fisher Scientific, Ottawa, ON, Canada) using Fluoromount (Sigma-Aldrich, Oakville, ON, Canada).

Neutrophil migration was quantified in corneal sections using an Axiovert 200M microscope with a Hamamatsu Orca R2 Camera (Zeiss, Thornwood, NY, USA). Three representative images (imaged at 20 × magnification) were taken from each section, corresponding to the right and left corneal peripheries and from the center of the cornea. Neutrophils from these three images were counted and summed to represent the total neutrophil per section. A total of 6–8 sections with 120 µm intervals were analyzed from each sample and were averaged. For each experimental group, 4–7 eyes were analyzed.

### 4.5. Pharmacological Treatments

The CB1 allosteric ligands GAT211, GAT228, and GAT229 were synthesized and provided by Ganesh A. Thakur (Northeastern University) [34]. Δ^8^-THC (Cayman Chemical, Ann Arbor, MI, USA), GAT211, GAT228, and GAT229 were dissolved in soybean oil with 2% dimethyl sulfoxide (DMSO; Sigma-Aldrich, Oakville, ON, Canada) and 4% Tween-20 (Sigma-Aldrich, Oakville, ON, Canada). Drugs or vehicle were topically administered (5 µL) to cauterized corneas at 30, 60, and 120 min post-cauterization. The CB1 antagonist AM251 (Tocris Bioscience, Minneapolis, MN, USA) was suspended in 10% DMSO in saline. AM251 was injected at a dose of 2.0 mg/kg i.p. 15 min before cauterization. Capsaicin (1 µM; Tocris Bioscience, Minneapolis, MN, USA) was prepared in DMSO diluted with sterile saline to 0.002%.

### 4.6. Data Analysis

Statistical analysis was performed in GraphPad Prism version 6. Unless otherwise indicated, one-way analysis of variance (ANOVA) with Dunnett’s post hoc was used to compare data between groups of three or more, while analysis between two groups was performed using *t*-test. All data are represented as group mean ± standard deviation and were considered significant at *p* < 0.05.
